# Effects of *Carissa opaca* fruits extracts on oxidative pulmonary damages and fibrosis in rats

**DOI:** 10.1186/1472-6882-14-40

**Published:** 2014-01-30

**Authors:** Sumaira Sahreen, Muhammad Rashid Khan, Rahmat Ali Khan

**Affiliations:** 1 Botanical Sciences Division, Pakistan Museum of Natural History, Garden Avenue, Shakarparian, Islamabad, Pakistan; 2 Department of Biotechnology, University of Science and Technology, Bannu, KPK, Pakistan; 3 Department of Biotechnology, Faculty of Biological Sciences, University of Science and Technology, Bannu, KPK 28100, Pakistan

**Keywords:** Carissa opaca, Lungs, CCl4, Antioxidant enzymes, TBARS, DNA

## Abstract

**Background:**

*Carissa opaca* is a Pakistani fruit, traditionally used in the treatment of various human ailments including asthma and pulmonary damage. The present study investigated the protective effects of *Carissa opaca* against CCl_4_-induced oxidative stress in rat lungs.

**Methods:**

To assess the protective effects of *Carissa opaca*, 42 Sprague–Dawley male rats (170–180 g) were randomly divided into 7 groups. Group I was untreated and group II received olive oil intraperitoneally (i.p.) and dimethyl sulfoxide orally. Groups III, IV, V, VI and VII were administered CCl_4_, 3 ml/kg bodyweight (30% in olive oil i.p.). Group IV was administered 50 mg/kg bodyweight silymarin whereas groups V, VI and VII were treated with 200 mg/kg of various fractions of *Carissa opaca* after 48 h of CCl_4_ treatment for eight weeks. Antioxidant profiles in lungs were evaluated by estimating the activities of antioxidant enzymes: catalase, peroxidase, superoxide dismutase, glutathione-S-transferase, glutathione reductase, glutathione peroxidase, quinone reductase and reduced glutathione. CCl_4_-induced lipid peroxidation was determined by measuring the level of thiobarbituric acid reactive substances (TBARS) with conjugation of DNA damage and histopathology.

**Results:**

Administration of CCl_4_ for 8 weeks significantly reduced *(p < 0.05)* the activities of antioxidant enzymes and GSH concentration while increasing TBARS content and DNA damage. Co-treatment of various fractions of *Carissa opaca* and silymarin restored the activities of antioxidant enzymes and glutathione content. Changes in TBARS concentration and DNA fragmentation was significantly decreased *(p < 0.05)* following *Carissa opaca* and silymarin treatment in lung.

**Conclusions:**

Histopathological changes in rat lungs induced by CCl_4_ were significantly restored by co-treatment with *Carissa opaca* and silymarin.

## Background

Exposure to UV radiation, X-rays, environmental pollutants, toxic chemicals and excessive drug use causes the production of free radicals and reactive oxygen species that lead to oxidative damage in the kidney, liver and lungs [1]. The lung is the main organ of respiration and is exposed to higher oxygen stress compared with other tissues. Exogenous oxidants, cigarette smoke and asbestos fibers activate inflammatory cells to generate oxidative stress and lung fibrosis [1]. Several important reactive oxygen species (ROS) are generated endogenously in these circumstances [2], which include superoxide radicals, hydrogen peroxide (H_2_O_2_), and hydroxyl radicals. The major enzymes/reaction pathways that are activated to generate ROS in human lungs include nicotinamide adenine dinucleotide phosphate oxidases, myeloperoxidase, eosinophil peroxidase, mitochondrial electron transport chain, and possibly xanthine oxidase [3]. A balance between intracellular and extracellular oxidants and antioxidants is a prerequisite for normal lung homeostasis. The lung has highly specialized and compartmentalized antioxidant defenses to protect against ROS and reactive nitrogen species. Induction of these antioxidant enzymes and related proteins after pulmonary insults may protect the lung and promote normal repair. Conversely, impaired induction or inactivation/clearance of antioxidant enzymes may result in a sustained redox imbalance that may contribute to the progression of pulmonary fibrosis [4].

Plants are commonly used for the discovery of new therapeutic products. In recent years, there has been increasing interest in finding natural antioxidants from commonly available wild plants, fruits and vegetables [5] as well as their important role in detoxification of free radical-induced lung injuries and fibrosis in experimental animal models [6]. *Carissa opaca* (*C. opaca*) Stapf ex Hanes is a 2–3 meter tall evergreen shrub containing glabrous fruits widely found in Pakistan [7]. Traditionally this plant is used for the treatment of asthma [8], hepatitis [9], diarrhea [10] and renal dysfunction [11]. The present study examined the toxic effects of CCl_4_ and investigated the beneficial effects of plant extracts on tissues from various CCl_4_-induced lung damage experimental groups.

## Methods and Materials

### Plant collection

*C. opaca* ripened fruits were collected in March-April 2011 from the Quaid-i-Azam University Islamabad, Pakistan. The plants were recognized by their local names and then validated by Dr. Mir Ajab Khan, Department of Plant Sciences, Quaid-i-Azam University, Islamabad. A voucher specimen with Accession No. 24561 (*C. opaca*) was deposited at the Herbarium of Pakistan Quaid-i-Azam University, Islamabad Pakistan.

### Extract preparation

The collected fruits were cleaned to get rid of dust particles and then dried under shade for one to two weeks. Willy Mill of 60-mesh size was used to prepare powder of dried samples. 5 kg of powdered sample was extracted twice with 10 L of 95% methanol at 25°C for 48 h. For filtration Whatman No. 1 filter paper was used and then filtrate was concentrated through rotary evaporator (Panchun Scientific Co., Kaohsiung, Taiwan) under reduced pressure at 40°C. In order to resolve the compounds with escalating polarity, a part of the extract was suspended in distilled water and subjected to liquid-liquid partition by using solvents in a sequence of n-hexane and ethyl acetate. After fractioning, the solvent of respective fractions was also evaporated by rotary evaporator. Extract was dried and then stored at 4°C for further *in vivo* investigation.

### Experimental plan

Six-week-old male Sprague Dawley rats weighing 180 ± 10 g were provided with food and water *ad libitum* and kept at 20-22°C on a 12-h light/dark cycle. All experimental procedures involving animals were conducted in accordance with the guidelines of National Institutes of Health (NIH guidelines). The study protocol were approved by Ethical committee of Quaid-i-Azam University Islamabad. The rats were acclimatized to laboratory condition for 7 days before commencement of experiment. For chronic toxicity eight week experiment was designed. 42 male albino rats were randomly divided into seven groups (6 rats of each group). Administration of CCl_4_ (0.5 ml/kg b.w., 20% CCl_4_/olive oil) was intraperitoneally (i.p.) twice a week for eight weeks. At the same time, the rats were administered individually silymarin (50 mg/kg b.w.) and extract (200 mg/kg b.w.) orally twice a week for eight weeks.

### Experimental protocol

Following dosing plan was adapted for the study.

Group I: Normal control received only feed

Group II: Olive oil (0.5 ml/kg b.w., i.p.) + DMSO (0.5 ml/kg b.w. orally)

Group III: CCl_4_ twice a week (0.5 ml/kg b.w., i.p., 20% CCl_4_/olive oil)

Group IV: CCl_4_ twice a week (0.5 ml/kg b.w., i.p.) + sylimarin (50 mg/kg b.w., orally)

Group V: CCl_4_ twice a week (0.5 ml/kg b.w., i.p.) + n-heaxne *Carissa opaca* fruit extract (HFC, 200 mg/kg b.w., orally)

Group VI: CCl_4_ twice a week (0.5 ml/kg b.w., i.p.) + Ethyl acetate *Carissa opaca* fruit extract (EFC 200 mg/kg b.w., orally)

Group VII: CCl_4_ twice a week (0.5 ml/kg b.w., i.p.) + Methanol *Carissa opaca* fruit extract (MFC 200 mg/kg b.w., orally)

At the end of eight weeks, after 24 h of the last treatment, animals were given chloroform anesthesia and dissected from ventral side. All the animals were sacrificed; lungs were removed and washed in ice cold saline. Subsequently, half of the organs were treated with liquid nitrogen and stored at -80°C for further enzymatic and DNA damage analysis while the other portion was processed for histology.

### Biochemical investigations

In order to evaluate the pharmacological effects of different fractions of *C. opaca* against the toxicity induced with CCl_4_ in rats following assays had been carried out.

### Assessment of antioxidant enzymes

10% tissue homogenate was prepared in 100 mM KH_2_PO_4_ buffer containing 1 mM EDTA (pH 7.4) and centrifuged at 12,000 × g for 30 min at 4°C. The supernatant was collected and used for the following parameters as described below.

### Catalase assay (CAT)

CAT activities were determined by the method of Chance and Maehly [12] with some modifications. The reaction solution of CAT activities contained: 2.5 ml of 50 mM phosphate buffer (pH 5.0), 0.4 ml of 5.9 mM H_2_O_2_ and 0.1 ml enzyme extract. Changes in absorbance of the reaction solution at 240 nm were determined after one minute. One unit of CAT activity was defined as an absorbance change of 0.01 as units/min.

### Peroxidase assay (POD)

Activities of POD were determined by the method of Chance and Maehly [12] with some modifications. The POD reaction solution contained: 2.5 ml of 50 mM phosphate buffer (pH 5.0), 0.1 ml of 20 mM guaiacol, 0.3 ml of 40 mM H_2_O_2_ and 0.1 ml enzyme extract. Changes in absorbance of the reaction solution at 470 nm were determined after one minute. One unit of POD activity was defined as an absorbance change of 0.01 units/min.

### Superoxide dismutase assay (SOD)

SOD activity was estimated by the method of Kakkar *et al*. [13]. Reaction mixture of this method contained: 0.1 ml of phenazine methosulphate (186 μM), 1.2 ml of sodium pyrophosphate buffer (0.052 mM; pH 7.0), 0.3 ml of supernatant after centrifugation (1500 × g for 10 min followed by 10000 × g for 15 min) of lung homogenate was added to the reaction mixture. Enzyme reaction was initiated by adding 0.2 ml of NADH (780 μM) and stopped after 1 min by adding 1 ml of glacial acetic acid. Amount of chromogen formed was measured by recording color intensity at 560 nm. Results are expressed in units/mg protein.

### Glutathione-S-transferase assay (GST)

Glutathione-S-transferase activity was assayed by the method of Habig *et al*. [14]. The reaction mixture consisted of 1.475 ml phosphate buffer (0.1 mol, pH 6.5), 0.2 ml reduced glutathione (1 mM), 0.025 ml (CDNB) (1 mM) and 0.3 ml of homogenate in a total volume of 2.0 ml. The changes in the absorbance were recorded at 340 nm and enzymes activity was calculated as nM CDNB conjugate formed/min/mg protein using a molar extinction coefficient of 9.6 × 10^3^ M^-1^ cm^-1^.

### Glutathione reductase assay (GR)

Glutathione reductase activity was determined by method of Carlberg and Mannervik [15]. The reaction mixture consisted of 1.65 ml phosphate buffer: (0.1 mol; pH 7.6), 0.1 ml EDTA (0.5 mM), 0.05 ml oxidized glutathione (1 mM), 0.1 ml NADPH (0.1 mmol) and 0.1 ml of homogenate in a total volume of 2 ml. Enzyme activity was quantitated at 25 ºC by measuring disappearance of NADPH at 340 nm and was calculated as nM NADPH oxidized/min/mg protein using molar extinction coefficient of 6.22 × 10^3^ M^-1^ cm^-1^.

### Glutathione peroxidase assay (GPx)

Glutathione peroxidase activity was assayed by the method of Mohandas *et al*. [16]. The reaction mixture consisted of 1.49 ml phosphate buffer (0.1 M; pH 7.4), 0.1 ml EDTA (1 mM), 0.1 ml sodium azide (1 mM), 0.05 ml glutathione reductase (1 IU/ml), 0.05 ml GSH (1 mM), 0.1 ml NADPH (0.2 mM), 0.01 ml H_2_O_2_ (0.25 mM) and 0.1 ml of homogenate in a total volume of 2 ml. The disappearance of NADPH at 340 nm was recorded at 25 ºC. Enzyme activity was calculated as nM NADPH oxidized/min/mg protein using molar extinction coefficient of 6.22 × 10^3^ M^-1^ cm^-1^.

### Quinone reductase assay (QR)

The activity of quinone reductase was determined by the method of Benson *et al*. [17]. The 3.0 ml reaction mixture consisted of 2.13 ml Tris-HCl buffer (25 mM; pH 7.4), 0.7 ml BSA, 0.1 ml FAD, 0.02 ml NADPH (0.1 mM), and 0.l ml of homogenate. The reduction of dichlorophenolindophenol (DCPIP) was recorded at 600 nm and enzyme activity was calculated as nM of DCPIP reduced/min/mg protein using molar extinction coefficient of 2.1 × 10^4^ M^-1^ cm^-1^

### Reduced glutathione assay (GSH)

Reduced glutathione was estimated by the method of Jollow *et al*. [18]. 1.0 ml sample of homogenate was precipitated with 1.0 ml of (4%) sulfosalicylic acid. The samples were kept at 4°C for 1 h and then centrifuged at 1200 × g for 20 min at 4°C. The total volume of 3.0 ml assay mixture contained 0.1 ml filtered aliquot, 2.7 ml phosphate buffer (0.1 M; pH 7.4) and 0.2 ml DTNB (100 mM). The yellow color developed was read immediately at 412 nm on a SmartSpecTM plus Spectrophotometer. It was expressed as μM GSH/g tissue.

### Estimation of lipid peroxidation assay (TBARS/LPO)

The assay for lipid peroxidation was carried out following the modified method of Iqbal *et al*. [19]. The reaction mixture in a total volume of 1.0 ml contained 0.58 ml phosphate buffer (0.1 M; pH 7.4), 0.2 ml homogenate sample, 0.2 ml ascorbic acid (100 mM), and 0.02 ml ferric chloride (100 mM). The reaction mixture was incubated at 37°C in a shaking water bath for 1 h. The reaction was stopped by addition of 1.0 ml 10% trichloroacetic acid. Following addition of 1.0 ml 0.67% thiobarbituric acid, all the tubes were placed in boiling water bath for 20 min and then shifted to crushed ice-bath before centrifuging at 2500 × g for 10 min. The amount of TBARS formed in each of the samples was assessed by measuring optical density of the supernatant at 535 nm using spectrophotometer against a reagent blank. The results were expressed as nM TBARS/min/mg tissue at 37°C using molar extinction coefficient of 1.56 × 10^5^ M^-1^ cm^-1^.

### Hydrogen peroxide assay (H_2_O_2_)

Hydrogen peroxide (H_2_O_2_) was assayed by H_2_O_2_-mediated horseradish peroxidase-dependent oxidation of phenol red by the method of Pick and Keisari [20]. 2.0 ml of homogenate sample was suspended in 1.0 ml of solution containing phenol red (0.28 nM), horse radish peroxidase (8.5 units), dextrose (5.5 nM) and phosphate buffer (0.05 M; pH 7.0) and were incubated at 37°C for 60 min. The reaction was stopped by the addition of 0.01 ml of NaOH (10 N) and then centrifuged at 800 × g for 5 min. The absorbance of the supernatant was recorded at 610 nm against a reagent blank. The quantity of H_2_O_2_ produced was expressed as nM H_2_O_2_/min/mg tissue based on the standard curve of H_2_O_2_ oxidized phenol red.

### Molecular studies

DNA had been isolated and its fragmentation percent was quantified in molecular studies of *in vivo* toxicity.

### DNA fragmentation assay with diphenylamine reaction

DNA fragmentation from tissue extract was determined using the procedure of Wu *et al*. [21]. 100 mg tissue was homogenized in TTE solution. 0.1 ml of homogenate was labeled B, centrifuged at 200 × g at 4°C for 10 min, got supernatant labeled S. S tubes were centrifuged at 20,000 × g for 10 min at 4°C to separate intact chromatin, was labeled T. 1.0 ml of 25% TCA was added in all tubes T, B, S and incubated over night at 4°C. After incubation precipitated DNA was recovered by pelleting for 10 min at 18,000 × g at 4°C. 160 μl of 5% TCA was added to each pellet and heated for 15 min at 90°C then 320 μl of freshly prepared DPA solution was added, vortexed and incubated for 4 hr 37°C. Optical density was read at 600 nm with a spectrophotometer (Smart spec™ Plus, catalog # 170-2525).

### DNA Isolations and ladder assay

DNA was isolated by using the methods of Wu *et al.*[21]. 100 mg of tissue in a petri dish was washed with DNA Buffer and homogenized in 1 ml lysis buffer. 100 μl of proteinase K (10 mg/ml) and 240 μl 10% SDS, shaked gently, and incubate overnight at 45°C in a water bath then 0.4 ml of phenol, was added shaked for 5-10 min, and centrifuge at 3000 rpm for 5 min at 10°C. Supernatant was mixed with 1.2 ml phenol, 1.2 ml chloroform/isoamyl alcohol (24:1); shaked for 5-10 min, and centrifuged at 3000 rpm for 5 min at 10°C. 25 μl of 3 M sodium acetate (pH 5.2) and 5 ml ethanol was added with supernatant, shake until DNA was precipitated. DNA was washed with 70% ethanol, dried, dissolved in TE buffer and its concentration checked at 260 and 280 nm.5 μg of total DNA and 0.5 μg DNA standard per well were loaded on 1.5% agarose gel containing ethidium bromide. Electrophoresis was performed for 45 min with 100 V batteries, and DNA was observed under digital gel doc system and photographed.

### Histopathological study of tissue

After weighting the portion specifies for histology small pieces of each tissue was fixed for 3-4 h in fixative sera followed by dehydration with ascending grades of alcohol (80%, 90%, and 100%) and transferred in cedar wood oil. When tissue becomes clear then all tissues were embedded in paraplast and prepared blocks for further microtomy. 3-4 μm thin slides were prepared with microtome; wax was removed, stained with hemotoxilin-eosin and photographed under light microscope at 10× and 40×.

### Statistical analysis

To find the different treatment effects, one way analysis of variance was carried by computer software SPSS 13.0. Level of significance among the various treatments was determined by LSD at 0.05% level of probability.

## Results

### Effects of *C. opaca* fruit against CCl_4_ induced pulmonary toxicity in rat

CCl_4_ is a powerful pulmonary toxin that induces acute and chronic lung toxicity. This study induced pulmonary toxicity by CCl_4_ and determined the therapeutic effect of *C. opaca* fruit in CCl_4_ administered rats. Parameters studied included antioxidant enzymatic levels, genotoxicity and characteristic histological findings of lungs.

### Effects of *C. opaca* fruit on enzymatic antioxidant levels

Oxidative stress can affect almost every organ including the lungs. ROS production is caused by oxidative stress that directly damages lipids, proteins and DNA. In reaction to this, the body has its own defense system that consists of antioxidant enzymes that limit the level of damage. The protective effects of different fractions of *C. opaca* fruit against CCl_4_-induced pulmonary changes of tissue proteins and antioxidant levels is shown in Table 1. CCl_4_ treatment considerably *(p < 0.05)* decreased tissue protein, catalase, peroxidase, and superoxide dismutase levels compared with the control group. Co-administration of EFC, HFC and MFC significantly *(p < 0.05)* increased antioxidant enzyme activity.

**Table 1:** **Effects of various fractions of *C. opaca *** **fruit on tissue proteins and antioxidant enzyme levels**

**Group**	**Protein (μg/mg tissue)**	**CAT (U/min)**	**POD (U/min)**	**SOD (U/mg protein)**
Control	2.01 ± 0.03f	4.11 ± 0.21d	10.42 ± 0.53d	3.40 ± 0.23e
Oil + DMSO	1.96 ± 0.03f	4.09 ± 0.30d	1I.00 ± 0.20d	3.25 ± 0.21e
CCl_4_	0.74 ± 0.01a	2.09 ± 0.24a	5.74 ± 0.42a	1.12 ± 0.02a
Sily + CCl_4_	1.49 ± 0.02e	3.60 ± 0.11c	9.64 ± 0.23c	2.59 ± 0.10d
HFC + CCl_4_	0.89 ± 0.03b	2.60 ± 0.16b	6.53 ± 0.32b	1.46 ± 0.10b
EFC + CCl_4_	1.05 ± 0.06c	3.02 ± 0.07c	6.89 ± 0.17b	1.51 ± 0.08b
MFC + CCl_4_	1.35 ± 0.078d	3.30 ± 0.10c	7.80 ± 0.17b	2.20 ± 0.13c

Values are Mean ± SD (06 number). Sily = Silymarin.

a-f (Means with different letters) indicate significance at p < 0.05.

### Effects of *C. opaca* fruit on TBARS and H_2_O_2_

The effects of *C. opaca* fruit on TBARS and H_2_O_2_ levels are shown in Table 2. Increased TBARS and H_2_O_2_ levels were due to CCl_4_ toxicity_._ Post-administration of various fractions of *C. opaca* fruits appreciably *(p < 0.05)* ameliorated the toxic effects of CCl_4_ in lung tissues in contrast to the control group. Similar ameliorating effects were observed with silymarin administration, which had levels similar to the control group.

**Table 2:** **Effects of various fractions of *C. opaca *** **fruit on tissue TBARS and H**_**2**_**O**_**2**_

**Group**	**TBARS (nM/min/mg protein)**	**H**_**2**_**O**_**2 **_**(nM/min/mg tissue)**
Control	3.13 ± 0.18c	1.21 ± 0.074f
Oil + DMSO	3.20 ± 0.13c	1.17 ± 0.051f
CCl_4_	5.41 ± 0.68a	2.64 ± 0.062a
Sily + CCl_4_	4.02 ± 0.47b	1.53 ± 0.058e
HFC + CCl_4_	4.91 ± 0.11a	2.24 ± 0.035b
EFC + CCl_4_	4.89 ± 0.15a	2.21 ± 0.043b
MFC + CCl_4_	4.07 ± 0.23b	1.99 ± 0.094c

Values are Mean ± SD (06 number). Sily = Silymarin.

a-f (Means with different letters) indicate significance at p < 0.05.

### Effects of *C. opaca* fruit on glutathione enzymes

Phase II antioxidant metabolizing enzymes play a key role in the detoxification of oxidative stress. CCl_4_ insults significantly *(p < 0.05)* reduced the activities of glutathione enzymes such as glutathione S-transferase (GST), glutathione peroxidase (GPx) and glutathione reductase (GR) when compared with the control group (Table 3). Various fractions of *C. opaca* fruits significantly *(p < 0.05)* ameliorated toxicity by increasing the activity of phase II antioxidant enzymes towards control group levels. Silymarin administration to rats significantly *(p < 0.05)* reduced toxicity and restored the activities of GST, GPx and GR in lung tissues.

**Table 3:** **Effects of various fractions of *C. opaca *** **fruit on GST, GPx and GSH**

**Group**	**GST (nM/min/mg protein)**	**GPx (nM/min/mgprotein)**	**GR (nM/min/mg protein)**
Control	170.29 ± 4.28 h	114.21 ± 3.29 g	247.01 ± 6.00 g
Oil + DMSO	177.43 ± 4.31 h	108.48 ± 3.12 g	236.23 ± 5.73 g
CCl_4_	92.13 ± 3.48a	65.08 ± 3.32a	136.24 ± 3.72a
Sily + CCl_4_	161.34 ± 3.22 g	101.48 ± 2.81f	192.41 ± 4.07e
HFC + CCl_4_	99.61 ± 2.03b	73.40 ± 1.45b	149.32 ± 4.56b
EFC + CCl_4_	105.34 ± 2.51c	72.02 ± 1.81b	154.66 ± 4.39b
MFC + CCl_4_	130.80 ± 3.73e	83.52 ± 2.40d	177.46 ± 4.29d

Values are Mean ± SD (06 number). Sily = Silymarin.

a-f (Means with different letters) indicate significance at p < 0.05.

### Effects of *C. opaca* fruit on GSH, QR and DNA fragmentation

Glutathiones and DNA play important roles in free radical-induced detoxification. Administration of CCl_4_ significantly reduced *(p < 0.05)* both GSH and QR content, and significantly *(p < 0.05)* increased DNA fragmentation (Table 4). Treatment of rats with various fractions of *C. opaca* significantly *(p < 0.05)* restored DNA fragmentation and GSH and QR activity compared with the CCl_4_ group. Silymarin also showed significant protection against CCl_4_.

**Table 4:** **Effects of various fractions of *C. opaca *** **fruit on GSH, QR and DNA fragmentation**

**Group**	**GSH (μM/g tissue)**	**QR (nM/min/mg protein)**	**DNA damages %**
Control	27.07 ± 1.41e	119.18 ± 3.03 g	12.82 ± 1.38e
Oil + DMSO	24.23 ± 1.50e	124.27 ± 3.68 g	13.53 ± 1.98e
CCl_4_	10.23 ± 0.65a	60.74 ± 2.13a	61.42 ± 2.70a
Sily + CCl_4_	20.53 ± 1.41d	104.47 ± 3.22f	19.19 ± 1.48d
HFC + CCl_4_	11.65 ± 0.46b	66.18 ± 2.87b	33.26 ± 0.72b
EFC + CCl_4_	11.90 ± 0.57b	73.30 ± 3.17c	33.47 ± 0.48b
MFC + CCl_4_	15.79 ± 1.00c	91.26 ± 2.32e	20.33 ± 1.54d

Values are Mean ± SD (06 number). Sily = Silymarin.

a-h (Means with different letters) indicate significance at p < 0.05.

### Effects of *C. opaca* fruit on DNA damages (ladder assay)

**Figure 1:**
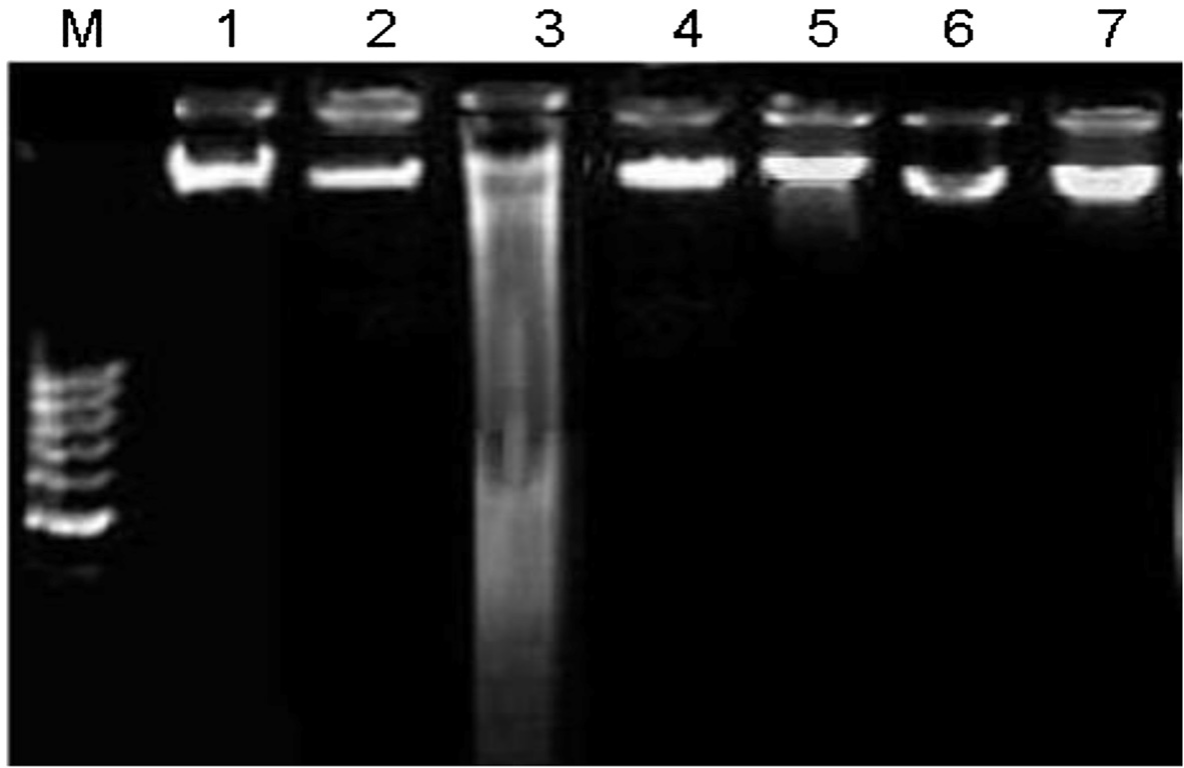
**Agarose gel showing DNA damage by CCl**_**4 **_**and protective effects of various fractions of *C. opaca *fruit in pulmonary tissue.** Lanes from left (M) low molecular weight marker, (1) control, (2) DMSO + Olive oil group, (3) CCl_4_ group, (4) Silymarin + CCl_4_ group, (5) MFC + CCl_4_ group, (6) EFC + CCl_4_ group, (7) HFC + CCl_4_ group.

CCl_4_ is a genotoxic chemical and causes DNA damages. The protective effects of various fractions of *C. opaca* fruit against CCl_4_ induced DNA damages in rats are shown by DNA ladder assay in Figure 1. Intact genomic DNA was revealed by ladder assay of control group while, CCl_4_ group showed marked DNA damages. Co-treatment of silymarin and various fractions viz; HFC, EFC and MFC proved the recovering effects by DNA band pattern showing similarity with control group.

### Effects of *C. opaca* fruit on lung histoarchitecture

**Figure 2:**
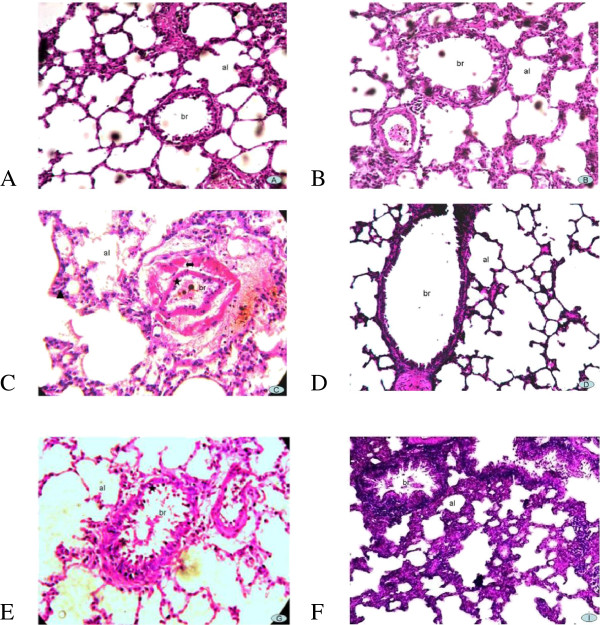
**Microphotograph of rat lungs (H & E stain) (A) Representative section of lungs from the control group showing normal histology, (B) DMSO + Olive oil group, (C) CCl**_**4 **_**group, (D) Silymarin + CCl**_**4 **_**group, (E) (E) MFC + CCl**_**4 **_**group, (F) EFC + CCl**_**4 **_**group.** (al) alveolar space, (br) bronchioles, (★) collapsed inner epithelial layer, () ruptured muscular layer with disorganized Clara cells, (▲) aggregation of fibroblasts.

The effects of various fractions of *C. opaca* fruits against CCl_4_ induced lung injury and histological changes were observed. Histological assessments of lung tissues of control and DMSO group confirmed the typical cellular architecture with distinct alveolar septa and bronchioles, structured Clara cells and fibroblasts as shown in Figure 2A and B, respectively. Aggregation of fibroblasts, collagen fibres, ruptured alveolar bronchioles and walls, disorganized Clara cells showing pulmonary edema and interstitial hemorrhage were found in CCl_4_ intoxicated rats (Figure 2C). The lung sections of rats treated with various fractions of *C. opaca* fruits viz; EFC and MFC intoxicated with CCl_4_ (Figure 2E-F), showed normal structure of alveolar bronchioles and less degenerative changes with various degrees in case of each group, supplementing the protective effects of the plant samples. Post-administrations of silymarin reduced the toxic effects of CCl_4_ and reversed the histopathology towards the control group (Figure 2D). Present histological observations are in agreement with the results of pulmonary oxidative stress level.

## Discussion

Lungs are the site of respiration and are exposed to the action of toxic chemicals, drugs or smoking. Toxin inhalation is often injurious to health and can cause pulmonary disease. Lungs are often injured by oxidative stress. A biochemical study suggested CCl_4_ free radicals could have the same consequences [2]. Pulmonary toxicity causes notable pathological effects such as fibrosis, inflammatory changes and degeneration of epithelial cells. Symptoms of pulmonary toxicity include fibrosis, inflammatory response, and degeneration of epithelial cells. Therefore, this study investigated the pharmacological outcomes of different plant extracts and their fractions on CCl_4_-induced oxidative damage in rat lungs [22]. The potency of natural antioxidants depends on their chain breaking capacity, declining hydrogen peroxide levels, scavenging superoxides and chelating transitory metal ions [23]. Food scientists and nutrition specialists suggest that plants are useful as a source of natural antioxidants that contribute to reducing risks of certain diseases, such as cancer and cardiovascular disease. In this context, intake of plants might prevent the onset of these diseases [24]. Food deterioration is brought about by lipid peroxidation. The use of synthetic drugs as preservative agents to inhibit lipid peroxidation are strictly prohibited as they are potent carcinogens [25]. Consequently, these chemical therapeutics/synthetic drugs should be replaced with naturally occurring agents having no or very few side-effects. A previous study verified that a diet high in vegetables and fruits is associated with a decline in degenerative diseases [26], thus natural antioxidants, flavonoids and phenolic compounds have gained considerable attention. Structurally, phenolic compounds contain a conjugated ring with a hydroxyl group, and can act as an antioxidant by preventing free radical-mediated diseases. Plant antioxidant capacity is characterized by quenching free radicals such as lipid peroxy radicals, singlet oxygen and superoxide anions [27]. Combinatorial methods are required to explore the antioxidant tendency of natural resources because no single assay can reflect all antioxidants in a mixed assay or the complex nature of phytochemicals. Adedapo et al. [28] reported the scavenging abilities of plant extracts against free radicals in a complex assay system to eradicate the radical-related pathological diseases. Toxic compounds of drugs and xenobiotics are metabolized by the glutathione system (reduced glutathione, glutathione reductase, glutathione peroxidase and glutathione-S-transferase). Administration of *C. opaca* reduced CCl_4_ toxicity, thereby increasing the activity of GST, GSR, GSH-Px and QR [29,30]. Similar observations were reported by Khan et al. [31], when administering melatonin against CCl_4_-induced oxidative stress. Free radicals cause lipid peroxidation, elevate TBARS and deplete tissue GSH contents [32]. In the present study, low levels of GSH were accompanied by elevated levels of TBARS and H_2_O_2_ compared with the control group. Fruit extracts of *C. opaca* were characterized by the high expression level of GSH contents with low level of TBARS and H_2_O_2_. Similar observations were reported during co-treatment of plant extracts against CCl_4_-induced damage in rats [33]. Lipid peroxidation induced by CCl_4_ disturbs protein synthesis but can also diffuse into the nucleus, causing DNA fragmentation [34,35] that can lead to pulmonary damages. In the present study, CCl_4_-induced DNA damage was significantly ameliorated by *C. opaca* as reported previously by Khan et al. [36]. Extensive variations were observed during histopathological study of rat lungs. CCl_4_ damage of the alveolar septa and mobbing of blood capillaries resulted in the accumulation of blood cells and collagen fibers at various places causing an endemic condition. Similar observations were found in rat lungs in previous studies during CCl_4_ administration [37]. Co-treatment with *C. opaca* repaired pulmonary damage, as demonstrated by normal spaces in the alveoli, reduced cellular degeneration of alveoli and bronchioles as well as normalized pneumocytes as previously reported by Khan et al. [38] during *Sonchus asper* administration against CCl_4_-induced injuries in rats.

## Conclusion

The present results revealed that *C. opaca* comprised of bioactive compounds; presenting protective effects against CCl_4_ induced toxic effects in lungs of rat. Further studies of isolation and purification of these constituents are in progress in our lab.

## Competing interests

The authors declare that they have no competing interests.

## Authors’ contributions

SS made significant contribution to acquisition of data, analysis, conception, design of the manuscript. MRK and RAK (ORCID ID: 0000-0003-0453-2090) made significant contribution to acquisition of data, analysis, drafting and conception. All the authors read and approved the final manuscript.

## Pre-publication history

The pre-publication history for this paper can be accessed here:

http://www.biomedcentral.com/1472-6882/14/40/prepub
